# Medical Students’ Vademecum

**Published:** 1852-05

**Authors:** 


MEDICAL STUDENTS' VADEMECUM.

This work is of the same character as the Compend of Drs. Neil and Smith, and is of established reputation, having passed to the third edition. Its accomplished author, Dr. Mendenhall, enriches each successive edition with the improvements in all the departments of medical science.

---

*State of Medical Science and of the Medical Profession in America.*—The great American Republic is claimed exultingly by Professor Henderson as the land of Homœopathy; and, as is well known, from that territory another Professor levies a large revenue for his pills and ointment. There too, in her "office" at New York, flourishes, as a consulting physician, Elizabeth Blackwell, M. D., "that unwomanly lady;" and there, in truth, quackery is rampant in its most subtile as well as in its grossest forms. There, till lately, the medical profession was in a chaotic state, and the line between regular and irregular practitioners was ill-defined; but a better era has now commenced. A vast effort is being made throughout the Union to raise the standard of medical character and medical education; and a sound ethical spirit is everywhere manifesting itself.—*London Journal of Medicine.*

